# Hydroxychloroquine Exposure Intensity During Early Pregnancy and Composite Fetal Adverse Pregnancy Outcomes in Systemic Lupus Erythematosus: A Retrospective Cohort Study

**DOI:** 10.3390/jcm15156121

**Published:** 2026-08-06

**Authors:** Meng Jiang, Yanling Chang, Jiayue Wu

**Affiliations:** 1 Department of Obstetrics and Gynecology, Renji Hospital, School of Medicine, Shanghai Jiao Tong University, Shanghai 200127, China; jiangmeng016369@renji.com (M.J.); changyanling@renji.com (Y.C.); 2 Shanghai Key Laboratory of Gynecologic Oncology, Shanghai 200127, China; 3 International Centre for Reproductive Health (ICRH), Department of Public Health and Primary Care, Faculty of Medicine and Health Sciences, Ghent University, 9000 Ghent, Belgium

**Keywords:** systemic lupus erythematosus, pregnancy, hydroxychloroquine, early pregnancy, exposure intensity, fetal adverse pregnancy outcomes, preterm birth

## Abstract

**Background**: Hydroxychloroquine (HCQ) is commonly recommended during pregnancy in women with systemic lupus erythematosus (SLE), but real-world patterns of early-pregnancy HCQ continuation, reduction, or interruption remain clinically relevant. This study aimed to evaluate the association between HCQ exposure intensity during early pregnancy and composite fetal adverse pregnancy outcomes in pregnancies complicated by SLE. **Methods**: This retrospective cohort study included 513 pregnancies complicated by SLE. HCQ exposure intensity during early pregnancy, defined as conception to 13 weeks and 6 days of gestation, was classified into three groups: no HCQ exposure, reduced/interrupted HCQ exposure, and full HCQ exposure. The primary outcome was composite fetal adverse pregnancy outcome, defined as fetal loss, preterm birth, or fetal growth restriction. Logistic regression models were used to estimate odds ratios (ORs) and 95% confidence intervals (CIs), with full HCQ exposure as the reference group. **Results**: Among 513 pregnancies, 96 (18.7%) had no HCQ exposure, 68 (13.3%) had reduced/interrupted HCQ exposure, and 349 (68.0%) had full HCQ exposure during early pregnancy. Composite fetal adverse pregnancy outcomes occurred in 47/96 (49.0%), 48/68 (70.6%), and 136/349 (39.0%) pregnancies, respectively (*p* < 0.001). After adjustment for maternal age, primiparity, lupus nephritis history, antiphospholipid antibody positivity, chronic hypertension, prednisone dose > 15 mg/day, and baseline immunosuppressant use, reduced/interrupted HCQ exposure was associated with higher odds of composite fetal adverse pregnancy outcome compared with full HCQ exposure (adjusted OR 4.17, 95% CI 2.25–7.75, *p* < 0.001). No independent association was observed for no HCQ exposure after adjustment (adjusted OR 1.30, 95% CI 0.78–2.16, *p* = 0.309). **Conclusions**: In pregnancies complicated by SLE, reduced or interrupted HCQ exposure during early pregnancy was independently associated with an increased risk of composite fetal adverse pregnancy outcomes, while no independent association was observed for no HCQ exposure after adjustment for baseline risk factors. These findings highlight the potential clinical importance of maintaining HCQ treatment continuity during early pregnancy.

## 1. Introduction

Systemic lupus erythematosus (SLE) often affects women during their reproductive years. Although pregnancy outcomes have improved with multidisciplinary care, SLE pregnancies still carry higher risks of fetal loss, preterm birth, fetal growth restriction (FGR), preeclampsia, and maternal complications than pregnancies in the general population [1,2]. The level of risk varies substantially between patients and is influenced by factors such as previous lupus nephritis, antiphospholipid antibody (aPL) positivity, chronic hypertension, and disease activity before or during pregnancy [2,3]. As a result, preconception assessment, stable disease control, and pregnancy-compatible treatment are central to clinical management [4,5]. Similar concerns regarding obstetric and fetal complications have also been reported in other high-risk reproductive populations, such as women with recurrent pregnancy loss [6].

Hydroxychloroquine (HCQ) is a foundational treatment for SLE. The updated EULAR recommendations for SLE management advise HCQ for patients with lupus unless contraindicated, and the drug is widely used to reduce disease activity, prevent flares, and limit glucocorticoid exposure [7,8]. In reproductive care, both EULAR and American College of Rheumatology recommendations support the use or continuation of pregnancy-compatible medications, including HCQ, in women with SLE [4,5].

The safety of HCQ in pregnancy is supported by a large body of observational evidence, but its effect on specific fetal and obstetric outcomes remains less settled. In the Hopkins lupus pregnancy cohort, women who discontinued HCQ in the 3 months before pregnancy or during the first trimester had higher lupus activity and flare rates than those who continued HCQ, without evidence of increased fetal toxicity [9]. An individual participant data meta-analysis of prospectively collected lupus pregnancies found lower odds of highly active lupus among HCQ users, but no statistically significant difference in fetal loss, preterm delivery, or preeclampsia overall; the authors also noted the challenges of confounding by indication and incomplete adherence information in observational studies [10]. More recently, a nationwide Swedish cohort reported that early-pregnancy HCQ exposure was associated with a lower risk of preeclampsia, while the association with preterm delivery remained uncertain [11].

Most prior studies have treated HCQ exposure as a binary variable. This approach is convenient, but it may miss clinically important patterns around pregnancy recognition. In routine care, some women have no HCQ exposure, some continue HCQ without interruption, and others reduce the dose, stop treatment, or have a period of interruption during early pregnancy. Reduced or interrupted exposure is not necessarily an intermediate state between non-use and full use. It may represent disruption of a treatment regimen that was already considered clinically relevant.

Early pregnancy is also a biologically meaningful window. Trophoblast invasion and spiral artery remodeling begin during this period and are essential for placental perfusion and fetal growth. Disturbances in these processes are implicated in placenta-mediated complications, including FGR and preeclampsia [12,13]. Whether the intensity and continuity of HCQ exposure during this window are associated with fetal outcomes in SLE pregnancies remains insufficiently characterized.

We therefore evaluated the association between HCQ exposure intensity during early pregnancy and fetal adverse pregnancy outcomes in a pregnancy-level cohort of SLE pregnancies. We classified exposure into three clinically interpretable groups—no exposure, reduced or interrupted exposure, and full exposure—and assessed their associations with fetal loss, preterm birth, FGR, and composite fetal adverse pregnancy outcome.

## 2. Materials and Methods

### 2.1. Study Design and Population

This retrospective pregnancy-level cohort study included pregnancies complicated by systemic lupus erythematosus (SLE) managed at Renji Hospital, School of Medicine, Shanghai Jiao Tong University, between February 2018 and June 2025. Eligible pregnancy records were identified from the institutional obstetric and rheumatology medical record systems.

Pregnancies were eligible for inclusion if they had a confirmed diagnosis of SLE and available information on hydroxychloroquine (HCQ) exposure during early pregnancy and pregnancy outcomes. After data review and removal of ineligible or duplicate records, 513 pregnancies were included in the final analysis. Each record corresponded to one pregnancy.

SLE diagnosis was based on clinical medical records and fulfilled the 2012 SLICC classification criteria [14]. The 2012 SLICC criteria were used because the cohort began in 2018, when these criteria were routinely used in clinical practice and research.

### 2.2. Definition of HCQ Exposure Intensity During Early Pregnancy

The primary exposure was HCQ exposure intensity during early pregnancy. Early pregnancy was defined as the period from conception to 13 weeks and 6 days of gestation, or to the occurrence of pregnancy loss if this occurred before 13 weeks and 6 days.

HCQ exposure information was extracted from outpatient prescriptions, inpatient records, medication documentation, and clinical notes. Pregnancies were classified into three mutually exclusive exposure groups according to documented HCQ use and treatment continuity during early pregnancy.

1. **No HCQ exposure**: No documented HCQ use before or during the early-pregnancy exposure window.
2. **Reduced or interrupted HCQ exposure**: Documented loss of HCQ treatment continuity during early pregnancy, including HCQ dose reduction, temporary interruption, or discontinuation recorded in prescriptions, medication documentation, or clinical notes. This category also included pregnancies in which HCQ had been used before conception but was discontinued or interrupted before or during the early-pregnancy assessment window. Therefore, the defining feature of this group was not simply partial exposure, but documented reduction or interruption of an established or intended HCQ regimen during the early-pregnancy period.
3. **Full HCQ exposure:** HCQ use during early pregnancy without documented dose reduction, discontinuation, or interruption. Pregnancies in which HCQ was initiated during early pregnancy and then continued without documented interruption were also classified as full exposure.

### 2.3. Outcomes

The primary outcome was composite fetal adverse pregnancy outcome. It was defined as the occurrence of at least one of the following: fetal loss, preterm birth, or fetal growth restriction (FGR). Fetal loss was defined according to obstetric records and included pregnancy loss or fetal demise. Preterm birth was defined as delivery before 37 completed weeks of gestation among live births. FGR was defined as an antenatal diagnosis documented in obstetric records, based on ultrasound evidence of impaired fetal growth according to institutional criteria.

Secondary outcomes included each component of the composite fetal outcome, live birth, gestational age at delivery, birth weight, preeclampsia, SLE activity during pregnancy, and renal involvement during pregnancy. Preeclampsia was analysed as a secondary obstetric outcome and was not included in the primary composite fetal outcome. SLE activity during pregnancy was analysed as a secondary disease-related outcome and was not included as an adjustment variable in the main model because it occurred after exposure classification during follow-up.

### 2.4. Covariates

Baseline demographic, obstetric, clinical, and treatment variables were extracted from medical records. Candidate baseline covariates included maternal age, primiparity, history of lupus nephritis, antiphospholipid antibody (aPL) positivity, antiphospholipid syndrome, chronic hypertension, prednisone dose > 15 mg/day before or during early pregnancy, baseline immunosuppressant use, 24 h proteinuria, and complement levels.

The main multivariable model adjusted for maternal age, primiparity, history of lupus nephritis, aPL positivity, chronic hypertension, prednisone dose > 15 mg/day, and baseline immunosuppressant use. These variables were selected based on clinical relevance and their potential roles as confounders of the association between HCQ exposure and fetal adverse pregnancy outcomes. Variables occurring during follow-up, including SLE activity during pregnancy, renal involvement during pregnancy, and pregnancy complications, were not included as covariates in the main model.

### 2.5. Statistical Analysis

Continuous variables were summarized as mean ± standard deviation or median with interquartile range, depending on distribution. Categorical variables were summarized as numbers and percentages. Baseline characteristics and pregnancy outcomes were compared across the three HCQ exposure groups using analysis of variance or the Kruskal–Wallis test for continuous variables and the chi-square test or Fisher’s exact test for categorical variables, as appropriate. For outcomes with an overall *p* value < 0.05 across the three HCQ exposure groups, pairwise post hoc comparisons were performed with Bonferroni correction for multiple comparisons. For categorical outcomes, pairwise Pearson chi-square tests or Fisher’s exact tests were used as appropriate, and Bonferroni-adjusted *p* values were calculated; for continuous outcomes, one-way analysis of variance with Bonferroni post hoc tests was used. Logistic regression models were used to evaluate the association between HCQ exposure intensity during early pregnancy and composite fetal adverse pregnancy outcome. The full HCQ exposure group was used as the reference category. Crude and adjusted odds ratios (ORs) with 95% confidence intervals (CIs) were reported.

Three models were constructed. The crude model included HCQ exposure group only. Model 1 adjusted for maternal age and primiparity. Model 2 further adjusted for history of lupus nephritis, aPL positivity, chronic hypertension, prednisone dose > 15 mg/day, and baseline immunosuppressant use. SLE activity during pregnancy was not included in the main multivariable model because it was assessed after HCQ exposure classification and may represent an intermediate variable on the pathway between HCQ treatment continuity and pregnancy outcomes. To avoid potential overadjustment, the main model adjusted for baseline clinical risk factors. As an exploratory sensitivity analysis, we additionally fitted a model including SLE activity during pregnancy to assess whether the association between HCQ exposure intensity and composite fetal adverse pregnancy outcome was materially changed. The same modelling strategy was used for selected secondary binary outcomes when event numbers were sufficient. Exploratory analyses were additionally performed for the individual components of the composite fetal adverse pregnancy outcome, including fetal loss, preterm birth, and FGR. Preterm birth was analysed among live births. Several supplementary analyses were performed. First, analyses were restricted to pregnancies with any HCQ exposure during early pregnancy, comparing reduced/interrupted HCQ exposure with full HCQ exposure. Second, SLE activity during pregnancy was analysed as a secondary disease-related outcome. Third, an additional model including laboratory variables, such as 24 h proteinuria and low complement levels, was explored when data completeness was acceptable.

All statistical tests were two-sided, and a *p* value < 0.05 was considered statistically significant. Statistical analyses were performed using SPSS version 29.0.

### 2.6. Ethics Approval

This retrospective cohort study protocol was initially approved by the Institutional Ethics Committee of Renji Hospital on 18 January 2018 (approval number: 2017-113), renewed on 25 December 2019 (approval number: KY2019-138), and further renewed on 20 February 2022 and 28 September 2025 (share same approval number: KY2021-199-B). Patients and the public were not involved in the design, conduct, reporting or dissemination plans of this retrospective study.

## 3. Results

### 3.1. Study Population and Baseline Characteristics

**Figure 1:**
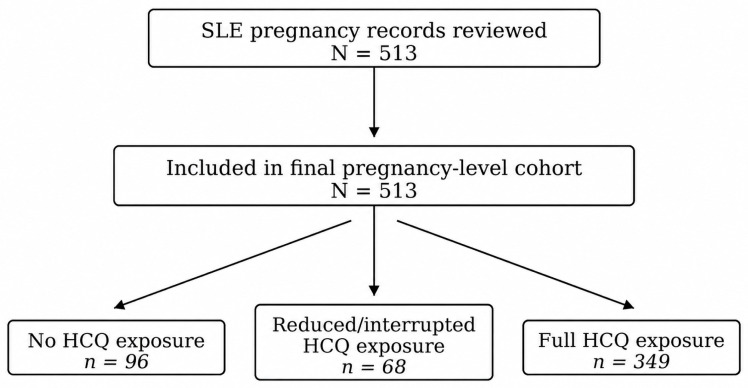
**Flowchart of the study population and HCQ exposure classification during early pregnancy.** A total of 513 SLE pregnancy records were included in the final pregnancy-level cohort and classified as no HCQ exposure (*n* = 96), reduced/interrupted HCQ exposure (*n* = 68), or full HCQ exposure (*n* = 349). HCQ, hydroxychloroquine; SLE, systemic lupus erythematosus.

A total of 513 pregnancies complicated by SLE were included in the final pregnancy-level cohort. During early pregnancy, 96 pregnancies (18.7%) had no HCQ exposure, 68 (13.3%) had reduced or interrupted HCQ exposure, and 349 (68.0%) had full HCQ exposure (Figure 1).

Baseline characteristics according to HCQ exposure intensity are shown in Table 1. Overall, the mean maternal age was 29.7 ± 4.0 years, and 238 pregnancies (46.4%) were primiparous. A history of lupus nephritis was present in 89 pregnancies (17.3%), aPL positivity in 94 (18.3%), chronic hypertension in 21 (4.1%), prednisone dose > 15 mg/day at baseline in 182 (35.5%), and baseline immunosuppressant use in 67 (13.1%). Baseline characteristics were generally comparable across the three HCQ exposure groups. No statistically significant differences were observed in maternal age, primiparity, history of lupus nephritis, aPL positivity, APS, chronic hypertension, prednisone dose > 15 mg/day, baseline immunosuppressant use, 24 h proteinuria, complement levels, or low complement status (Table 1).

### 3.2. Pregnancy Outcomes According to HCQ Exposure Intensity

**Figure 2:**
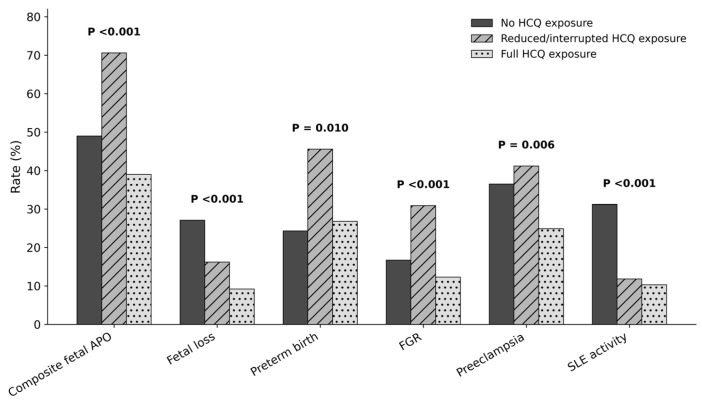
**Rates of composite fetal adverse pregnancy outcome and selected pregnancy and disease-related outcomes according to HCQ exposure intensity during early pregnancy.** Composite fetal APO was defined as fetal loss, preterm birth, or FGR. Preeclampsia was analysed as a secondary obstetric outcome and was not included in the composite fetal APO. *p* values were derived from chi-square tests or Fisher’s exact tests, as appropriate. APO, adverse pregnancy outcome; FGR, fetal growth restriction; HCQ, hydroxychloroquine; SLE, systemic lupus erythematosus.

Pregnancy outcomes according to HCQ exposure intensity are presented in Table 2. Overall, composite fetal adverse pregnancy outcome occurred in 231 of 513 pregnancies (45.0%). The frequency differed significantly across the three exposure groups: 47/96 (49.0%) in the no-exposure group, 48/68 (70.6%) in the reduced/interrupted exposure group, and 136/349 (39.0%) in the full-exposure group (*p* < 0.001) (Table 2 and Figure 2). In Bonferroni-adjusted post hoc pairwise comparisons, significant differences in composite fetal APO were observed between the no-exposure and reduced/interrupted groups and between the reduced/interrupted and full-exposure groups (Supplementary Table S4).

The individual components of the composite fetal outcome also varied across groups. Fetal loss occurred in 69 pregnancies (13.5%) overall and was most frequent in the no-exposure group (26/96, 27.1%), followed by the reduced/interrupted group (11/68, 16.2%) and the full-exposure group (32/349, 9.2%; *p* < 0.001). Among live births, preterm birth occurred in 128 of 444 pregnancies (28.8%) and was most frequent in the reduced/interrupted group (26/57, 45.6%), compared with the no-exposure group (17/70, 24.3%) and the full-exposure group (85/317, 26.8%; *p* = 0.010). FGR occurred in 80 pregnancies (15.6%) and was also most frequent in the reduced/interrupted group (21/68, 30.9%), compared with the no-exposure group (16/96, 16.7%) and the full-exposure group (43/349, 12.3%; *p* < 0.001) (Table 2 and Figure 2).

Live birth occurred in 444 pregnancies (86.5%) overall. The live birth rate was lowest in the no-exposure group (70/96, 72.9%) and highest in the full-exposure group (317/349, 90.8%; *p* < 0.001). Preeclampsia, analysed as a secondary obstetric outcome and not included in the primary composite fetal outcome, occurred in 150 pregnancies (29.2%) and differed across groups (35/96, 36.5%; 28/68, 41.2%; and 87/349, 24.9% in the no-exposure, reduced/interrupted, and full-exposure groups, respectively; *p* = 0.006).

SLE activity during pregnancy occurred in 74 pregnancies (14.4%) overall and also differed across groups. It was most frequent in the no-exposure group (30/96, 31.2%), compared with the reduced/interrupted group (8/68, 11.8%) and the full-exposure group (36/349, 10.3%; *p* < 0.001). Renal involvement during pregnancy was more frequent in the no-exposure and reduced/interrupted groups than in the full-exposure group (35/96, 36.5%; 28/68, 41.2%; and 83/349, 23.8%, respectively; *p* = 0.002).

Mean gestational age at delivery was 35.0 ± 6.3 weeks overall and differed significantly across groups (33.3 ± 7.5 weeks, 34.1 ± 6.3 weeks, and 35.7 ± 5.8 weeks in the no-exposure, reduced/interrupted, and full-exposure groups, respectively; *p* = 0.002). Among live births, mean birth weight was 2785 ± 502 g overall and was lowest in the reduced/interrupted group (2586 ± 587 g), compared with the no-exposure group (2752 ± 533 g) and the full-exposure group (2828 ± 470 g; *p* = 0.003). Bonferroni-adjusted pairwise comparisons are summarized in Supplementary Table S4. For composite fetal APO, significant differences were observed between the no-exposure and reduced/interrupted groups and between the reduced/interrupted and full-exposure groups. For secondary outcomes, pairwise differences showed distinct outcome patterns across the three exposure groups, with detailed adjusted *p* values provided in Supplementary Table S4.

### 3.3. Association Between HCQ Exposure Intensity and Composite Fetal Adverse Pregnancy Outcome

Logistic regression analyses evaluating the association between HCQ exposure intensity and composite fetal adverse pregnancy outcome are shown in Table 3. Full HCQ exposure was used as the reference group. In the crude model, reduced/interrupted HCQ exposure was associated with higher odds of composite fetal adverse pregnancy outcome compared with full HCQ exposure (OR 3.76, 95% CI 2.14–6.61, *p* < 0.001). No HCQ exposure showed a non-significant trend toward higher odds of the primary outcome compared with full exposure (OR 1.50, 95% CI 0.95–2.37, *p* = 0.079) (Table 3).

After adjustment for maternal age and primiparity, reduced/interrupted HCQ exposure remained associated with increased odds of composite fetal adverse pregnancy outcome (Model 1: OR 3.87, 95% CI 2.19–6.83, *p* < 0.001), whereas no HCQ exposure remained non-significant (Model 1: OR 1.49, 95% CI 0.94–2.35, *p* = 0.090) (Table 3).

**Figure 3:**
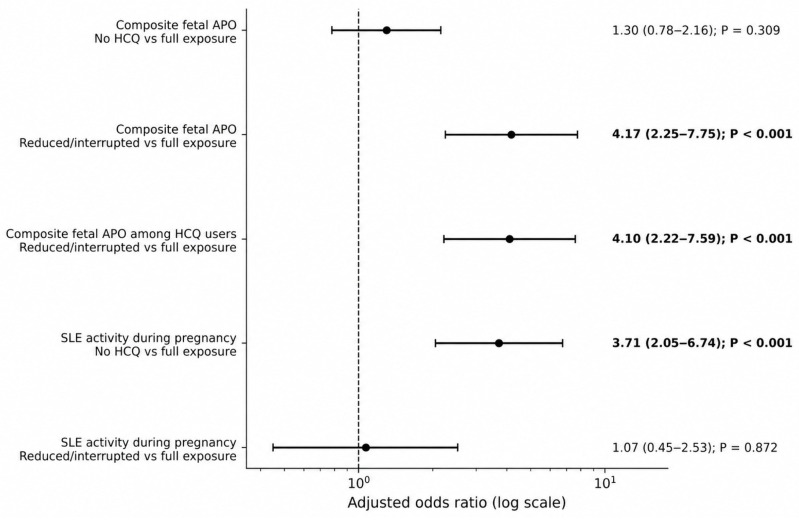
**Adjusted associations of HCQ exposure intensity with composite fetal adverse pregnancy outcome and SLE activity during pregnancy.** Odds ratios were estimated using multivariable logistic regression. For composite fetal APO, the main model adjusted for maternal age, primiparity, history of lupus nephritis, aPL positivity, chronic hypertension, prednisone > 15 mg/day, and baseline immunosuppressant use. The analysis restricted to HCQ-exposed pregnancies compared reduced/interrupted HCQ exposure with full HCQ exposure. For SLE activity during pregnancy, the model adjusted for maternal age, history of lupus nephritis, aPL positivity, chronic hypertension, prednisone > 15 mg/day, and baseline immunosuppressant use. Points indicate adjusted ORs; horizontal lines indicate 95% CIs; the dashed vertical line indicates OR = 1. APO, adverse pregnancy outcome; CI, confidence interval; FGR, fetal growth restriction; HCQ, hydroxychloroquine; OR, odds ratio; SLE, systemic lupus erythematosus.

In the main multivariable model, which further adjusted for history of lupus nephritis, aPL positivity, chronic hypertension, prednisone dose > 15 mg/day at baseline, and baseline immunosuppressant use, reduced/interrupted HCQ exposure remained independently associated with higher odds of composite fetal adverse pregnancy outcome compared with full HCQ exposure (Model 2: adjusted OR 4.17, 95% CI 2.25–7.75, *p* < 0.001). In contrast, no independent association was observed for no HCQ exposure after multivariable adjustment (Model 2: adjusted OR 1.30, 95% CI 0.78–2.16, *p* = 0.309) (Table 3 and Figure 3).

The full multivariable model is shown in Supplementary Table S1. In this model, chronic hypertension and prednisone dose > 15 mg/day at baseline were also independently associated with composite fetal adverse pregnancy outcome. Chronic hypertension was associated with increased odds of the primary outcome (adjusted OR 5.97, 95% CI 1.58–22.62, *p* = 0.009), and prednisone dose > 15 mg/day at baseline was associated with more than fivefold higher odds of the primary outcome (adjusted OR 5.26, 95% CI 3.44–8.04, *p* < 0.001) (Supplementary Table S1).

### 3.4. Supplementary and Secondary Analyses

Supplementary and secondary analyses are summarized in Supplementary Tables S2 and S3 and Figure 3. In the analysis restricted to pregnancies with any HCQ exposure during early pregnancy, reduced/interrupted HCQ exposure remained associated with higher odds of composite fetal adverse pregnancy outcome compared with full exposure (adjusted OR 4.10, 95% CI 2.22–7.59, *p* < 0.001). In contrast, when HCQ exposure was analysed as a binary variable, any HCQ exposure was not significantly associated with composite fetal adverse pregnancy outcome after multivariable adjustment.

Additional adjustment for 24 h proteinuria and low complement levels yielded consistent results. In an exploratory model additionally including SLE activity during pregnancy, reduced/interrupted HCQ exposure remained strongly associated with composite fetal adverse pregnancy outcome compared with full HCQ exposure (adjusted OR = 4.23, 95% CI 2.27–7.89, *p* < 0.001). No significant association was observed for no HCQ exposure after this additional adjustment (adjusted OR = 1.07, 95% CI 0.63–1.83, *p* = 0.798). SLE activity during pregnancy was also associated with composite fetal adverse pregnancy outcome in this exploratory model (adjusted OR = 2.94, 95% CI 1.58–5.47, *p* < 0.001) (Supplementary Table S2). For the disease-related secondary outcome, no HCQ exposure was associated with higher odds of SLE activity during pregnancy, whereas reduced/interrupted HCQ exposure was not significantly associated with this outcome (Supplementary Table S2).

In exploratory analyses of the individual components of the composite fetal adverse pregnancy outcome, different outcome patterns were observed. No HCQ exposure was associated with higher odds of fetal loss compared with full HCQ exposure (adjusted OR 3.41, 95% CI 1.77–6.57, *p* < 0.001), whereas reduced/interrupted HCQ exposure was not significantly associated with fetal loss. Among live births, reduced/interrupted HCQ exposure was associated with higher odds of preterm birth (adjusted OR 2.16, 95% CI 1.15–4.08, *p* = 0.017), whereas no significant association was observed for no HCQ exposure. Reduced/interrupted HCQ exposure was also associated with higher odds of FGR (adjusted OR 3.24, 95% CI 1.73–6.08, *p* < 0.001), whereas no HCQ exposure was not significantly associated with FGR (Supplementary Table S3).

## 4. Discussion

In this retrospective pregnancy-level cohort of 513 pregnancies complicated by SLE, HCQ exposure intensity during early pregnancy was associated with composite fetal adverse pregnancy outcomes. The highest frequency of composite fetal APO was observed among pregnancies with reduced or interrupted HCQ exposure, followed by those with no HCQ exposure and those with full HCQ exposure. After adjustment for baseline clinical risk factors, reduced or interrupted HCQ exposure remained independently associated with a markedly increased risk of composite fetal APO, whereas no independent association was observed for no HCQ exposure. This finding suggests that HCQ treatment continuity during early pregnancy may be more clinically informative than a simple exposed versus unexposed classification.

At first glance, it may appear counterintuitive that reduced or interrupted HCQ exposure was independently associated with composite fetal APO, whereas no independent association was observed for no HCQ exposure. However, these two exposure groups likely represent different clinical populations rather than simple ordered levels of the same exposure. Pregnancies with no HCQ exposure may include women with milder disease, women without a clear prior indication for HCQ, or women managed without HCQ because of stable disease or other clinical considerations. In such cases, the crude association between no exposure and fetal outcomes may be explained by baseline risk factors such as lupus nephritis, chronic hypertension, glucocorticoid exposure, and immunosuppressant use. This interpretation is consistent with the attenuation of the association for no HCQ exposure after multivariable adjustment. In contrast, reduced or interrupted exposure identifies pregnancies in which HCQ was considered clinically relevant or had already been used, but treatment continuity was not maintained during early pregnancy. By definition, the reduced/interrupted exposure group captured documented loss of HCQ treatment continuity during early pregnancy, including dose reduction, temporary interruption, or discontinuation of a previously established or intended HCQ regimen. This pattern may reflect disruption of an established disease-control regimen, poor adherence, medication intolerance, safety concerns, or changes in clinical management. Therefore, reduced or interrupted HCQ exposure should not be interpreted simply as an intermediate category between non-use and full use; rather, it may represent a distinct high-risk treatment pattern. Importantly, this finding should not be interpreted as evidence that no HCQ exposure is harmless, nor as proof that HCQ reduction or interruption directly causes fetal adverse outcomes. Instead, it suggests that early-pregnancy HCQ treatment continuity may provide clinically relevant risk information beyond a simple exposed-versus-unexposed classification.

Potential confounding by SLE disease activity should be carefully considered when interpreting these findings. We did not include SLE activity during pregnancy in the main model because it was assessed after HCQ exposure classification and may partly represent an intermediate variable rather than a pure baseline confounder. Instead, the main model was adjusted for baseline clinical factors that reflect underlying disease severity or pregnancy risk, including lupus nephritis history, aPL positivity, chronic hypertension, prednisone dose, and baseline immunosuppressant use. In an exploratory model additionally including SLE activity during pregnancy, reduced/interrupted HCQ exposure remained strongly associated with composite fetal adverse pregnancy outcome, and the effect estimate was not materially changed. This finding supports the robustness of the primary association, although residual confounding by disease activity cannot be fully excluded in this retrospective observational study.

This interpretation is supported by evidence showing that HCQ maintenance and discontinuation are not equivalent clinical states in SLE. In a randomized withdrawal trial in quiescent SLE, patients who continued HCQ were less likely to experience clinical flare-ups than those in whom HCQ was withdrawn [15]. Subsequent cohort studies also support the clinical relevance of HCQ treatment continuity. The SLICC inception cohort showed that HCQ tapering and discontinuation were associated with a higher risk of subsequent SLE flare compared with HCQ maintenance [16]. In another SLICC analysis using serum HCQ concentrations, severe non-adherence to HCQ was independently associated with increased risks of SLE flare, early damage, and mortality [17]. These data support the concept that interruption or non-continuity of HCQ therapy can carry clinically meaningful information beyond a simple classification of exposed versus unexposed.

Pregnancy-specific evidence also supports careful attention to HCQ continuation, while acknowledging that its effects on fetal and obstetric outcomes are not fully consistent across studies. In the Hopkins lupus pregnancy cohort, discontinuation of HCQ in the 3 months before pregnancy or during the first trimester was associated with higher lupus activity and flare rates, without evidence of fetal toxicity from HCQ exposure [9]. An individual participant data meta-analysis of prospectively collected lupus pregnancies found that HCQ use was associated with lower odds of highly active lupus, but not with statistically different odds of fetal loss, preterm delivery, or preeclampsia overall [10]. A systematic review and meta-analysis focusing on FGR and prematurity in lupus pregnancy did not prove a clear protective effect of HCQ for these outcomes [18], whereas another meta-analysis suggested that HCQ treatment during pregnancy may reduce the risks of preeclampsia, gestational hypertension, and prematurity [19]. These mixed findings are consistent with our binary HCQ analysis, in which any HCQ exposure was not independently associated with composite fetal APO. Our three-level exposure classification may therefore provide additional clinical information by separating full exposure from reduced or interrupted exposure.

The association between HCQ exposure intensity and composite fetal APO showed different patterns across individual components. In exploratory component analyses, no HCQ exposure was mainly associated with fetal loss, whereas reduced/interrupted HCQ exposure was associated with preterm birth and FGR. This pattern suggests that the composite outcome was not driven by a single component, but it also indicates that different HCQ exposure patterns may be associated with different types of fetal adverse outcomes. These outcomes are biologically plausible in SLE pregnancies, in which fetal outcomes are influenced by maternal disease status, lupus nephritis, aPL positivity, chronic hypertension, glucocorticoid exposure, placental dysfunction, and obstetric decision-making [1,2,3,4,20,21]. The need for careful reproductive risk assessment is also supported by studies of women with rheumatic diseases undergoing assisted reproductive technologies [22]. Early pregnancy is a key window for placental development, trophoblast invasion, and uterine spiral artery remodeling; abnormalities in these processes are implicated in placental-mediated complications, including FGR and preeclampsia [12,13,23]. Although the present study cannot establish a causal effect of HCQ interruption on placental development, the timing of exposure assessment during early pregnancy is clinically relevant and supports further investigation into whether maintaining HCQ exposure during this period may contribute to improved fetal outcomes.

Our results also highlight why exposure definition matters in observational studies of medication use during pregnancy. A simple binary comparison of HCQ users and non-users may combine pregnancies with full HCQ exposure and those with reduced or interrupted exposure into a single exposed group, thereby diluting differences related to treatment continuity. This issue is particularly relevant because observational studies of HCQ in lupus pregnancy are vulnerable to confounding by indication, incomplete adherence information, and differences in baseline disease severity [10]. Recent work using measured HCQ levels during pregnancy further suggests that subtherapeutic or non-adherent HCQ levels in the first trimester are associated with severe maternal flares, although not necessarily with obstetric or fetal outcomes [24]. These findings support the need to distinguish prescribed exposure, actual adherence, and treatment continuity in future studies.

It is noteworthy that no HCQ exposure was associated with SLE activity during pregnancy in secondary analyses, whereas reduced/interrupted HCQ exposure was not significantly associated with this disease-related outcome. This may reflect heterogeneity in the no-exposure group and differences between clinically documented maternal disease activity and fetal outcomes. Fetal APOs in SLE are multifactorial and may be influenced by chronic vascular risk, renal history, aPL-related mechanisms, glucocorticoid exposure, placental dysfunction, and timing of obstetric intervention, not only by overt lupus activity recorded during pregnancy. Therefore, the association between HCQ exposure intensity and fetal outcomes may not be fully mediated by clinically documented SLE activity in routine medical records.

The clinical implication of this study is not that no HCQ exposure is harmless, nor that HCQ interruption directly causes fetal adverse outcomes. Rather, our findings suggest that HCQ treatment continuity during early pregnancy deserves careful attention in SLE pregnancy management. For women already receiving HCQ before or during early pregnancy, dose reduction or interruption should be avoided unless there is a clear clinical indication. Concerns about fetal safety should be addressed through preconception counselling and early pregnancy education, because international recommendations support HCQ use before conception and throughout pregnancy in women with SLE [4,5]. Available systematic reviews of HCQ exposure in pregnancy have generally supported fetal safety, although residual confounding by disease activity should be considered [25].

This study has several strengths. First, HCQ exposure was classified into three clinically meaningful categories rather than as a simple binary variable, allowing assessment of treatment continuity during early pregnancy. Second, the primary outcome was defined as a fetal composite outcome excluding preeclampsia, thereby focusing on fetal loss, preterm birth, and FGR. Third, multivariable models adjusted for major baseline clinical risk factors, including lupus nephritis history, aPL positivity, chronic hypertension, glucocorticoid exposure, and baseline immunosuppressant use. Additional analyses restricted to pregnancies with any HCQ exposure and models including laboratory variables showed consistent results.

Several limitations should also be acknowledged. First, this was a retrospective single-center study, and residual confounding cannot be excluded. The reduced/interrupted exposure group may differ from the full exposure group in unmeasured ways, including disease duration, adherence behavior, socioeconomic factors, physician recommendations, and reasons for HCQ modification. Residual confounding by disease activity remains an important limitation. Although the main model was adjusted for several baseline indicators of clinical risk, including lupus nephritis history, glucocorticoid exposure, immunosuppressant use, aPL positivity, and chronic hypertension, we could not fully capture disease duration, preconception disease activity, physician decision-making, medication adherence, or the reasons for HCQ modification. In addition, SLE activity during pregnancy was not included in the main model because it occurred after exposure classification and may partly mediate the relationship between HCQ treatment continuity and pregnancy outcomes. Although the association between reduced/interrupted HCQ exposure and composite fetal adverse pregnancy outcome remained robust in an exploratory model additionally adjusting for SLE activity during pregnancy, the observed association should still be interpreted as an association rather than evidence of causality. Second, HCQ exposure was based on medical records and medication documentation rather than drug blood levels; therefore, actual adherence could not be objectively confirmed. Third, the reasons for HCQ reduction or interruption were not systematically captured, limiting our ability to distinguish physician-directed dose modification from patient-initiated discontinuation or intermittent non-adherence. Fourth, SLE activity during pregnancy was based on clinical documentation rather than a uniform validated disease activity index for all pregnancies. Finally, because this was an observational study, the findings should be interpreted as associations and not as proof of causality.

In conclusion, reduced or interrupted HCQ exposure during early pregnancy was independently associated with an increased risk of composite fetal adverse pregnancy outcomes in pregnancies complicated by SLE, whereas no independent association was observed for no HCQ exposure after adjustment for baseline risk factors. These findings support the clinical importance of evaluating HCQ exposure intensity and maintaining treatment continuity during early pregnancy, rather than relying solely on binary HCQ exposure classification.

## 5. Conclusions

In this retrospective pregnancy-level cohort of pregnancies complicated by SLE, reduced or interrupted HCQ exposure during early pregnancy was independently associated with a higher risk of composite fetal adverse pregnancy outcomes, including fetal loss, preterm birth, and FGR. In contrast, no independent association was observed for no HCQ exposure after adjustment for baseline clinical risk factors. These findings suggest that early-pregnancy HCQ treatment continuity may provide clinically relevant information beyond a simple exposed versus unexposed classification. For women with SLE who are receiving HCQ before or during early pregnancy, avoiding unnecessary dose reduction or interruption may be an important component of pregnancy management. Further prospective studies are needed to confirm these findings and to clarify the mechanisms linking HCQ treatment continuity with fetal outcomes in SLE pregnancies.

## Figures and Tables

**Table 1:** Baseline characteristics according to HCQ exposure intensity during early pregnancy.

Characteristic	Overall (N = 513)	No Exposure (N = 96)	Reduced/Interrupted (N = 68)	Full Exposure (N = 349)	*p* Value
Maternal age, years	29.7 ± 4.0	28.9 ± 4.1	30.0 ± 3.7	29.9 ± 4.0	0.079
Primiparity	238/513 (46.4)	47/96 (49.0)	34/68 (50.0)	157/349 (45.0)	0.642
History of lupus nephritis	89/513 (17.3)	22/96 (22.9)	15/68 (22.1)	52/349 (14.9)	0.101
aPL positivity	94/513 (18.3)	20/96 (20.8)	13/68 (19.1)	61/349 (17.5)	0.741
APS	19/512 (3.7)	3/96 (3.1)	1/67 (1.5)	15/349 (4.3)	0.509
Chronic hypertension	21/513 (4.1)	2/96 (2.1)	6/68 (8.8)	13/349 (3.7)	0.083
Prednisone >15 mg/day at baseline	182/513 (35.5)	42/96 (43.8)	26/68 (38.2)	114/349 (32.7)	0.116
Baseline immunosuppressant use	67/513 (13.1)	15/96 (15.6)	12/68 (17.6)	40/349 (11.5)	0.272
24 h proteinuria, g	0.14 (0.00–0.47)	0.18 (0.00–1.13)	0.16 (0.00–0.88)	0.12 (0.00–0.37)	0.059
C3, g/L	1.01 (0.84–1.18)	0.98 (0.83–1.15)	0.97 (0.75–1.18)	1.02 (0.86–1.19)	0.274
Low C3	156/513 (30.4)	31/96 (32.3)	24/68 (35.3)	101/349 (28.9)	0.526
C4, g/L	0.16 (0.11–0.22)	0.15 (0.10–0.22)	0.17 (0.11–0.21)	0.17 (0.12–0.22)	0.627
Low C4	83/513 (16.2)	20/96 (20.8)	14/68 (20.6)	49/349 (14.0)	0.158

Values are n/N (%) unless otherwise indicated. Continuous variables are shown as mean ± SD or median (IQR), as appropriate. aPL, antiphospholipid antibody; APS, antiphospholipid syndrome; HCQ, hydroxychloroquine; IQR, interquartile range; SD, standard deviation.

**Table 2:** Pregnancy outcomes according to HCQ exposure intensity during early pregnancy.

Outcome	Overall (N = 513)	No Exposure (N = 96)	Reduced/Interrupted (N = 68)	Full Exposure (N = 349)	*p* Value
Composite fetal APO	231/513 (45.0)	47/96 (49.0)	48/68 (70.6)	136/349 (39.0)	**<0.001**
Fetal loss	69/513 (13.5)	26/96 (27.1)	11/68 (16.2)	32/349 (9.2)	**<0.001**
Preterm birth	128/444 (28.8)	17/70 (24.3)	26/57 (45.6)	85/317 (26.8)	**0.010**
Fetal growth restriction	80/513 (15.6)	16/96 (16.7)	21/68 (30.9)	43/349 (12.3)	**<0.001**
Live birth	444/513 (86.5)	70/96 (72.9)	57/68 (83.8)	317/349 (90.8)	**<0.001**
Preeclampsia	150/513 (29.2)	35/96 (36.5)	28/68 (41.2)	87/349 (24.9)	**0.006**
SLE activity during pregnancy	74/513 (14.4)	30/96 (31.2)	8/68 (11.8)	36/349 (10.3)	**<0.001**
Renal involvement during pregnancy	146/513 (28.5)	35/96 (36.5)	28/68 (41.2)	83/349 (23.8)	**0.002**
Gestational age at delivery, weeks	35.0 ± 6.3	33.3 ± 7.5	34.1 ± 6.3	35.7 ± 5.8	**0.002**
Birth weight among live births, g	2785 ± 502	2752 ± 533	2586 ± 587	2828 ± 470	**0.003**

Composite fetal APO was defined as fetal loss, preterm birth, or FGR. Preeclampsia was analysed as a secondary obstetric outcome and was not included in the composite fetal APO. Values are n/N (%) unless otherwise indicated. *p* values < 0.05 are shown in bold. APO, adverse pregnancy outcome; FGR, fetal growth restriction; HCQ, hydroxychloroquine; SLE, systemic lupus erythematosus.

**Table 3:** Association between HCQ exposure intensity and composite fetal adverse pregnancy outcome.

Exposure	Crude OR (95% CI)	*p* Value	Model 1 OR (95% CI)	*p* Value	Model 2 OR (95% CI)	*p* Value
No HCQ exposure vs. full exposure	1.50 (0.95–2.37)	0.079	1.49 (0.94–2.35)	0.090	1.30 (0.78–2.16)	0.309
Reduced/interrupted HCQ exposure vs. full exposure	**3.76 (2.14–6.61)**	**<0.001**	**3.87 (2.19–6.83)**	**<0.001**	**4.17 (2.25–7.75)**	**<0.001**

Full HCQ exposure was the reference group. Model 1 adjusted for maternal age and primiparity. Model 2 adjusted for maternal age, primiparity, history of lupus nephritis, aPL positivity, chronic hypertension, prednisone > 15 mg/day, and baseline immunosuppressant use. *p* values < 0.05 are shown in bold; statistically significant ORs are also shown in bold. CI, confidence interval; HCQ, hydroxychloroquine; OR, odds ratio.

## Data Availability

The data presented in this study are available from the corresponding author upon reasonable request. The data are not publicly available due to patient privacy and institutional ethical restrictions.

## Supplementary Materials

The following supporting information can be downloaded at: https://www.mdpi.com/article/10.3390/jcm15156121/s1. Table S1: Full multivariable model for composite fetal adverse pregnancy outcome; Table S2: Sensitivity and secondary analyses; Table S3: Exploratory analyses of individual components of the composite fetal adverse pregnancy outcome; Table S4: Bonferroni-adjusted pairwise comparisons of pregnancy outcomes among HCQ exposure groups.
