# Supplementary material for: Hydroxychloroquine Exposure Intensity During Early Pregnancy and Composite Fetal Adverse Pregnancy Outcomes in Systemic Lupus Erythematosus: A Retrospective Cohort Study
Source: J Clin Med. 2026 Aug 6;15(15):6121. doi: 10.3390/jcm15156121 (PMC13466505; doi:10.3390/jcm15156121)
Supplement: Supplementary file 1 [file jcm-15-06121-s001.zip › jcm-4414386-supplementary.pdf]

Supplementary Table S1. Full multivariable model for composite fetal adverse pregnancy outcome

Variable	Adjusted OR (95% CI)	P value
No HCQ exposure vs full exposure	1.30 (0.78–2.16)	0.309
Reduced/interrupted HCQ exposure vs full exposure	**4.17 (2.25–7.75)**	**<0.001**
Maternal age, per year	1.01 (0.96–1.06)	0.735
Primiparity	0.79 (0.53–1.19)	0.263
History of lupus nephritis	1.60 (0.93–2.74)	0.089
aPL positivity	1.42 (0.85–2.36)	0.185
Chronic hypertension	**5.97 (1.58–22.62)**	**0.009**
Prednisone >15 mg/day at baseline	**5.26 (3.44–8.04)**	**<0.001**
Baseline immunosuppressant use	1.56 (0.85–2.85)	0.152

The dependent variable was composite fetal APO. Full HCQ exposure was the reference group. The model adjusted for all variables shown. P values <0.05 are shown in bold; statistically significant ORs are also shown in bold. APO, adverse pregnancy outcome; CI, confidence interval; HCQ, hydroxychloroquine; OR, odds ratio.

Supplementary Table S2. Sensitivity and secondary analyses

Analysis	Comparison	Outcome	Adjusted OR (95% CI)	P value	N
HCQ-exposed pregnancies only	Reduced/interrupted vs full HCQ exposure	Composite fetal APO	**4.10 (2.22–7.59)**	**<0.001**	417
Binary HCQ exposure	Any HCQ exposure vs no HCQ exposure	Composite fetal APO	0.96 (0.58–1.57)	0.868	513
Additional adjustment for proteinuria and complement	No HCQ exposure vs full exposure	Composite fetal APO	1.18 (0.69–2.02)	0.539	513
Additional adjustment for proteinuria and complement	Reduced/interrupted vs full HCQ exposure	Composite fetal APO	**4.03 (2.15–7.53)**	**<0.001**	513
Exploratory adjustment for SLE activity during pregnancy	No HCQ exposure vs full exposure	Composite fetal APO	1.07 (0.63–1.83)	0.798	513
Exploratory adjustment for SLE activity during pregnancy	Reduced/interrupted vs full HCQ exposure	Composite fetal APO	**4.23 (2.27–7.89)**	**<0.001**	513
Exploratory adjustment for SLE activity during pregnancy	SLE activity during pregnancy	Composite fetal APO	**2.94 (1.58–5.47)**	**<0.001**	513
Disease-related secondary outcome	No HCQ exposure vs full exposure	SLE activity during pregnancy	**3.71 (2.05–6.74)**	**<0.001**	513
Disease-related secondary outcome	Reduced/interrupted vs full HCQ exposure	SLE activity during pregnancy	1.07 (0.45–2.53)	0.872	513

All models adjusted for the same baseline covariates as the main model unless otherwise specified; the exploratory model additionally included SLE activity during pregnancy. The disease-related secondary outcome model did not include primiparity. Significant ORs and P values are shown in bold. APO, adverse pregnancy outcome; CI, confidence interval; HCQ, hydroxychloroquine; OR, odds ratio; SLE, systemic lupus erythematosus.

Supplementary Table S3. Exploratory analyses of individual components of the composite fetal adverse pregnancy outcome

Outcome	Comparison	Adjusted OR (95% CI)	P value	N	Events
Fetal loss	No HCQ exposure vs full exposure	**3.41 (1.77–6.57)**	**<0.001**	513	69
Fetal loss	Reduced/interrupted HCQ exposure vs full exposure	1.46 (0.62–3.43)	0.381	513	69
Preterm birth among live births	No HCQ exposure vs full exposure	0.82 (0.43–1.57)	0.553	444	128
Preterm birth among live births	Reduced/interrupted HCQ exposure vs full exposure	**2.16 (1.15–4.08)**	**0.017**	444	128
FGR	No HCQ exposure vs full exposure	1.24 (0.65–2.38)	0.511	513	80
FGR	Reduced/interrupted HCQ exposure vs full exposure	**3.24 (1.73–6.08)**	**<0.001**	513	80

All models adjusted for maternal age, primiparity, history of lupus nephritis, aPL positivity, chronic hypertension, prednisone >15 mg/day, and baseline immunosuppressant use. Full HCQ exposure was used as the reference group. Preterm birth was analysed among live births. Significant ORs and P values are shown in bold. CI, confidence interval; FGR, fetal growth restriction; HCQ, hydroxychloroquine; OR, odds ratio.

Supplementary Table S4. Bonferroni-adjusted pairwise comparisons of pregnancy outcomes among HCQ exposure groups

Outcome	Pairwise comparison	Adjusted p value
Composite fetal APO	No exposure vs reduced/interrupted exposure	**0.017**
	Reduced/interrupted exposure vs full exposure	**<0.001**
Fetal loss	No exposure vs full exposure	**<0.001**
Preterm birth among live births	No exposure vs reduced/interrupted exposure	**0.035**
	Reduced/interrupted exposure vs full exposure	**0.013**
FGR	Reduced/interrupted exposure vs full exposure	**<0.001**
Live birth	No exposure vs full exposure	**<0.001**
Preeclampsia	Reduced/interrupted exposure vs full exposure	**0.018**
SLE activity during pregnancy	No exposure vs full exposure	**<0.001**
	No exposure vs reduced/interrupted exposure	**0.011**
Renal involvement during pregnancy	No exposure vs full exposure	**0.038**
	Reduced/interrupted exposure vs full exposure	**0.009**
Gestational age at delivery, weeks	No exposure vs full exposure	**0.002**
Birth weight among live births, g	Reduced/interrupted exposure vs full exposure	**0.002**

Only significant Bonferroni-adjusted pairwise differences are shown for outcomes with an overall p value <0.05 across the three HCQ exposure groups. APO, adverse pregnancy outcome; FGR, fetal growth restriction; HCQ, hydroxychloroquine; SLE, systemic lupus erythematosus.
